# A study to identify individuals at risk to be affected by late-onset Pompe disease who had previously been given a non-specific or tentative diagnosis for their muscle weakness (Pompe PURSUE)

**DOI:** 10.1186/s13023-024-03425-1

**Published:** 2025-01-14

**Authors:** Dawn A. Laney, Kayla A. Banks, Eleanor G. Botha, Maria Keever, Valynne Long, Allison L. Foley

**Affiliations:** 1 Department of Human Genetics, Emory University, Atlanta, GA USA; 2 Parent-Project Muscular Dystrophy, Washington, DC USA; 3 WakeMed Physician Practices, Raleigh, NC USA

**Keywords:** Risk assessment, Genetic testing, Complex disease, Late-onset Pompe disease, Electronic medical record, Acid-α-glucosidase, Glycogen storage disease type II, Acid maltase deficiency, Undiagnosed patients, Limb-girdle muscular dystrophy, Symptom-based scoring tool

## Abstract

**Background:**

Late-onset Pompe disease (LOPD) is an autosomal recessive lysosomal storage disorder that results in severe progressive proximal muscle weakness. Over time, reductions in muscle strength result in respiratory failure and a loss of ambulation. Delayed diagnosis of LOPD deprives patients of treatments that can enhance quality of life and potentially slow disease progression. The objective of this study is to determine if patients with a nonspecific diagnosis, such as muscle weakness, may be at risk for LOPD using retrospective chart review of patients seen at two centers: an academic center and a community health system.

**Results:**

Initial data pulls identified 80,070 patients with one of the ICD-10 codes of interest. Chart review found 551 of these patients also had at least one lab value commonly abnormal in individuals with LOPD and of these 110 scored as “at-risk”. After removing phenocopies/other confirmed unrelated diagnoses, 46 individuals were contacted either directly or through their healthcare provider for genetic counseling. Three patients had pretest genetic counseling and were tested for decreased levels of acid-α-glucosidase. One patient was found to have deficient acid-α-glucosidase. Additionally, a physician educated through the program ordered LOPD testing for their patient and diagnosed them with LOPD.

**Conclusion:**

This study confirms that a symptom-based scoring tool and chart review combined with provider education can identify patients who are at increased likelihood to have a missed LOPD diagnosis.

**Supplementary Information:**

The online version contains supplementary material available at 10.1186/s13023-024-03425-1.

## Introduction

Pompe disease (PD), also known as glycogen storage disease type II and acid maltase deficiency, is an autosomal recessive progressive muscle disorder [1]. The condition is caused by deficiency of acid-α-glucosidase (GAA) associated with pathogenic variants in the *GAA* gene [1]. Deficiency of GAA results in the accumulation of glycogen in muscle tissues leading to irreversible muscle damage and a range of clinical symptoms [1, 2]. PD symptoms occur on a spectrum, but are typically categorized into two broad forms, infantile and late-onset. Within the same form of PD patients can have a variable progression of the disease, involvement of body tissues, and presentation of symptoms [1–3].

Late-onset Pompe disease (LOPD) is generally defined as presenting after the first year of life [2, 3]. Symptoms of LOPD typically include weakness in trunk and pelvic muscles, high creatine kinase levels, and respiratory insufficiency. The average time from symptom onset to diagnosis of LOPD has been found to be as long as ten years [2–5]. When a patient has been misdiagnosed with another neuromuscular disease the delay in correct diagnosis of LOPD can be prolonged by 2.5 to 10.5 years [3–6]. Delayed diagnosis of LOPD is due in part to overlapping symptoms that exist with other neuromuscular diseases [4–6]. For example, LOPD presents similarly to many of the limb girdle muscular dystrophies (LGMD) where pelvic and proximal muscles are weaker than distal arm and leg muscles [4–6].

In 2006, treating PD became a possibility with the first FDA licensing of an enzyme replacement therapy (ERT) for PD followed by specific licensing approval for use in LOPD in 2010 [7, 8]. Additional products to treat PD have since entered the market. Although ERT cannot cure LOPD, it has the potential to help stabilize a patient’s condition and slow the progression of the disease, allowing for a better quality of life [7, 8]. A review of ERT by Hagemans et al. showed that for each year a patient with LOPD goes untreated after diagnosis, the likelihood of wheelchair requirement increases by thirteen percent and the need for either invasive or non-invasive ventilation increases by eight percent [8]. Therefore, it is imperative to identify patients efficiently and provide them with the greatest possible quality of life.

Most research suggests that the combined incidence of both infantile and late-onset PD is about 1:40,000 [9]. It is believed that LOPD is more common than the infantile form due to the increased likelihood of pathogenic variants with milder phenotypes to be passed through generations. In 2011, a research study on newborn screening in Taiwan found the incidence of LOPD to be 1:26,466 [10]. In Austria, a newborn screening study found an incidence of LOPD to be as high as 1:8,684 [11]. In 2013, Missouri was the first state in the United States to implement newborn screening for PD. Over the six years, 36 infants were diagnosed with LOPD for an approximate incidence of 1: 14,567 [12]. In Georgia, a state pilot screening project on 59,332 newborns found 2 cases of confirmed LOPD for an incidence of 1 in 29,666 [13].

Prior to our study, there were fifteen known adult cases of LOPD in Georgia based on information from the Emory Lysosomal Storage Disease Center that theoretically receives all referrals for LOPD patients in the state and none treated at Lafayette General Hospital System (Now Ochsner Lafayette General Medical Center). However, using the conservative incidence estimate of 1:29,666, approximately 125 people in Georgia should be living with LOPD, suggesting significant under diagnosis of this treatable condition.

Efforts have been made to establish guidelines to shorten the time to LOPD diagnosis. In 2009, the American Association of Neuromuscular and Electrodiagnostic Medicine established an algorithm where GAA enzyme testing should be considered when a patient presents with limb girdle weakness, axial weakness of the paraspinal muscles, mild scapular winging, and symptoms of orthopnea [2, 3]. Vissing et al. encouraged the utilization of GAA enzyme assay as a first tier test and urged physicians to conduct retrospective chart reviews of patients presenting with elevated creatine kinase levels and inconclusive limb girdle muscle weakness to rule out LOPD [2].

In 2013, Preisler et al. conducted the first retrospective chart review looking for patients with undetected LOPD [6]. The authors identified 3 of 103 patients with LOPD, all of whom had elevated creatine kinase and unclassified LGMD diagnosis [6]. This study focused on two neuromuscular centers and one respiratory center to recruit possible participants. Preisler et al. selected participants that had elevated creatine kinase, unexplained myopathy on muscle biopsy, unexplained respiratory insufficiency, or unspecified myopathy. In 2013, Keever et al. completed a retrospective chart review looking for patients with undetected LOPD using the ICD-9 code code 359.1 in a neuromuscular center at Emory Healthcare [14]. They identified 637 patients that led to the genetic counseling and testing of 23 subjects. In the end, 2 patients and several additional family members (1 sibling and 2 children) were diagnosed with LOPD [14]. In 2023, a group in Spain took a slightly different approach by reviewing all adult internal medicine patients seen in 13 hospitals who presented with: polymyositis or any type of myopathy of unknown etiology; diagnosis of obstructive sleep apnea syndrome by polysomnography together with a body mass index (BMI) ≤ 30 kg/m^2^; asymptomatic or pauci-symptomatic elevated creatine kinase. After dried blood spot testing for GAA enzyme and confirmatory molecular testing of the *GAA* gene, they found 3/322 individuals were affected by LOPD [15].

The overall objective of this study was to build on this literature by expanding the scope of high-risk patient review to all patients seen in two very different health care systems: a large academic medical center in Georgia and a small regional hospital in the underserved medical region of Louisiana. The study goal was to find patients at-risk for LOPD via Electronic Medical Records (EMRs) data review starting with an automated data pull for specific ICD-10 codes and then utilizing a symptom-based scoring tool using observational clinical information, laboratory results, and billing information.

## Methods

### EMR data description

Prior to any study activities being performed, Emory University Institutional Review Board approval was obtained for this study (eIRB approval: IRB00114703). The study began with a retrospective EMR data pull of patients over age 18 years and coded with ICD-10 codes G71.0 (Proximal muscle weakness/muscular dystrophy), E74.00 (Glycogen storage disease, unspecified), E74.02 (Pompe disease), or E74.09 (Other glycogen storage disease) AND a list of individuals with elevated liver functions (Aspartate transaminase/AST ≥ 39 U/L and/or Alanine transaminase/ALT ≥ 52 U/L) or creatinine kinase (CK) values ≥ 223 U/L seen in the outpatient or inpatient population covered by Emory Healthcare between June 1, 2019 and June 1, 2020 with a total of 12,215,763 patients with EMRs available during this period. The Emory Healthcare system, located in the broader Atlanta, Georgia area, includes 11 hospitals, the outpatient Emory Clinic specialty and primary care clinics, a lysosomal storage disease center, and more than 250 individual provider locations covering patients primarily in Georgia. An additional existing data set of outpatient or inpatient population seen at Lafayette General Hospital System (LGH), now Ochsner Lafayette General Medical Center, from January 1, 2021- December 31, 2021 with a total of 242,000 patients with EMRs available for review was also queried for the same values. At the time of the study, LGH was a community teaching hospital that including a full-service medical center with associated outpatient specialties in the Lafayette, Louisiana region covering the section of southern central Louisiana between Baton Rouge, Louisiana and Beaumont, Texas. These two geologically diverse sets of medical records were chosen to determine if at-risk patients could be identified in two very different medical settings who may prioritize coding and laboratory testing in different ways. Additionally, prior to this study, there were no patients diagnosed with LOPD identified at LGH and no medical genetics within the hospital system. It was hypothesized that there might be a higher likelihood of finding patients at-risk for LOPD that had not been evaluated in this dataset.

### Validated symptom-based scoring tool description

The symptom-based scoring tool used in this study was developed, validated, and published by key members of the study team prior to this study. As described in Laney et al., 2022, the scoring system began with a review of the published literature to understand the frequency and specificity of key clinical features in LOPD [1–7, 14, 18] Very strong indicators, such as glycogen storage on muscle tissue, were scored as 100 points. Strong indicators, such as limb girdle (proximal) muscle weakness were scored 50 points. Moderate indicators, such as scapular winging, were scored with 24 points (Table 1). A score of 100 points or higher placed patients in the “at-risk” category who merited further follow-up. Then the scoring system was tested and modified using positive control patients already diagnosed with Pompe disease [18]. The final model had a tested sensitivity of 95.4% and a specificity of 100% in the original diagnosed data set i [18]. The binary nature of the tool allowed each chart to only have been reviewed once with minimal, if any, discrepancies in determination if a patient scored at or above 100 points (Table 1).

**Table 1:** Late onset Pompe disease symptom-based scoring tool using an at-risk cut off of 100 points

Indicators	Family history	Systemic	Symptoms
Very strong (score = 100 points)	A1) Sibling with PD	A2) Coded as unspecified glycogen storage disease (E74.00 glycogen storage, unspecified or E74.09 other glycogen storage disease)	
		A3) Glycogen storage on biospy/positive protein testing immunostaining on muscle tissue (may be coded as E74.00 glycogen storage, unspecified or E74.09 other glycogen storage disease)	
Strong (score = 50 points)	B1) First degree relative with PD	B3) Limb girdle (proximal) muscle weakness (G71.0 muscular dystrophy)	B5) Orthopnea (difficulty in breathing while lying down)
	B2) First degree relative with muscle weakness	B4) Any diagnosis of muscular dystrophy	B6) Respiratory insufficiency
			B7) Progressive proximal muscular weakness
			B8) Elevated creatine kinase (CK) levels
			B9) Muscle weakness (non irritable)
			B10) Gower maneuver
Moderate (score = 25 points)	C1) Consanguineous parents		C2) Scapular winging
			C3) Normal cardiac function
			C4) Morning headaches

In this study, after the data pull of patients who had an ICD-10 code of interest and elevated ALT, AST, or CK values, the study team then did a preliminary data review all of the patients to look for any other explanations of the findings such as biliary atresia, non-alcoholic fatty liver disease, a stroke, or a cancer diagnosis. The remaining patients were then scored using the validated symptom-based scoring tool (Table 1). The tool provided a streamlined way to calculate a patient “score” based on the symptoms, family history, and assessments found when reviewing patient records.

### Provider and patient contact

After ruling out any confirmed phenocopies/other genetic diagnoses again, the “at-risk” patients were contacted either directly or through their health care provider to participate further in the study. All healthcare providers were given information on the scoring tool, the diagnosis, how to refer patients to genetics, and how to test for LOPD. Patients referred by their physician or contacted directly by the study team that were interested in being tested for LOPD went through a detailed informed consent process in person or via telephone. Patients who consented to participate in the study had the option to come to clinic for a blood draw or to be tested locally. A sample of whole blood was taken from participants and GAA enzyme assay was performed through The Lantern Project, a complimentary genetic testing program sponsored by Sanofi partnered with PerkinElmer Genomics. Any samples with reduced GAA enzyme levels (< 3.88pmo) and enough remaining sample, had reflex *GAA* gene sequencing analysis (with copy number variant analysis as appropriate) to confirm LOPD.

## Results

The retrospective EMR data pulls from both institutions found 80, 070 patients (from the total record set of 12,215,763 patients at Emory Healthcare and 242,000 at LGH over age 18 years and coded with ICD-10 codes G71.0, E74.00, E74.02, or E74.09. A separate list of individuals with elevated liver functions (Aspartate transaminase/AST and/or Alanine transaminase/ALT) or creatinine kinase (CK) values was also provided by data pull to narrow down to the “at-risk” study population.

**Fig. 1:**
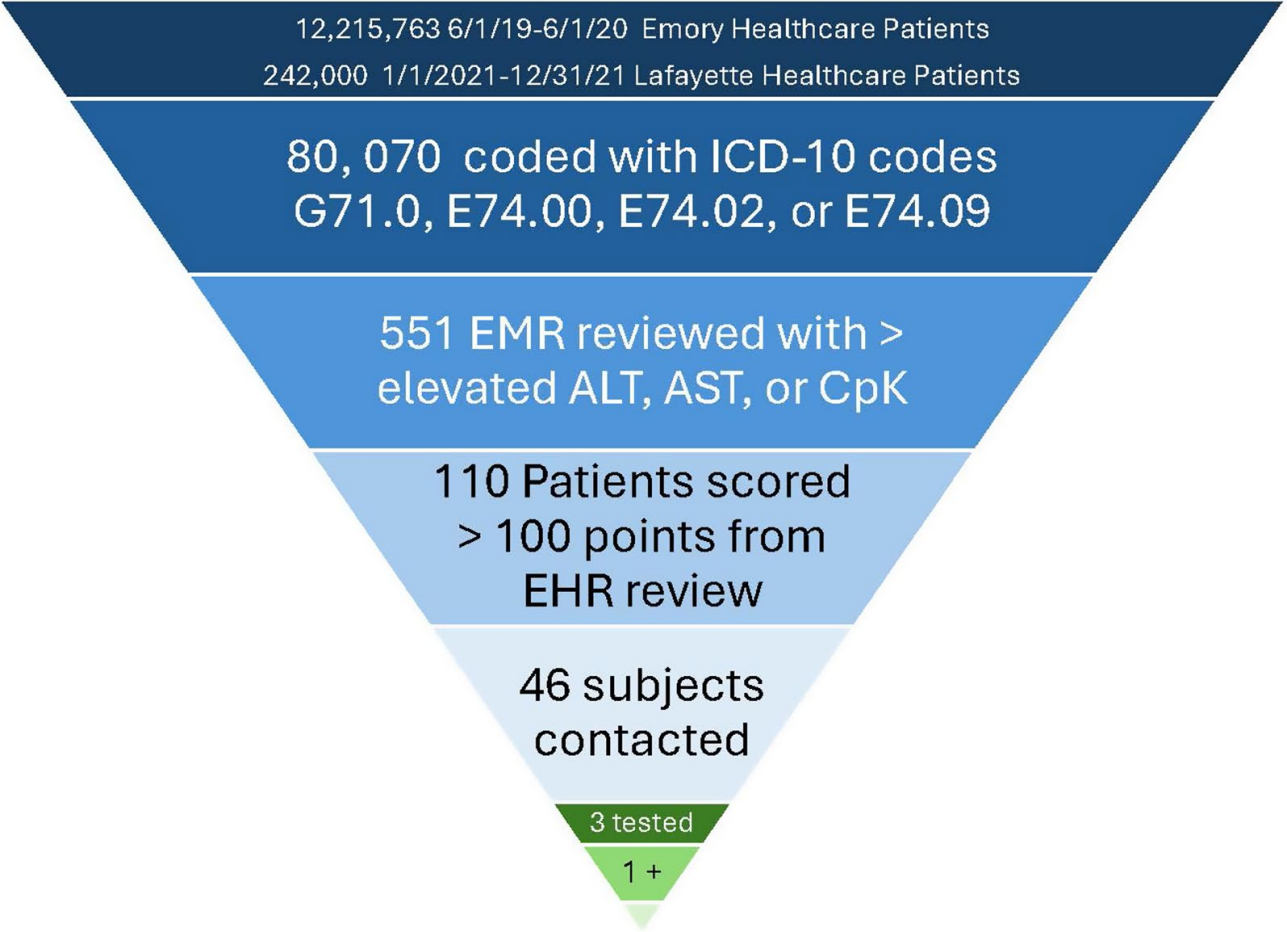
Study participant recruitment

**Fig. 2:**
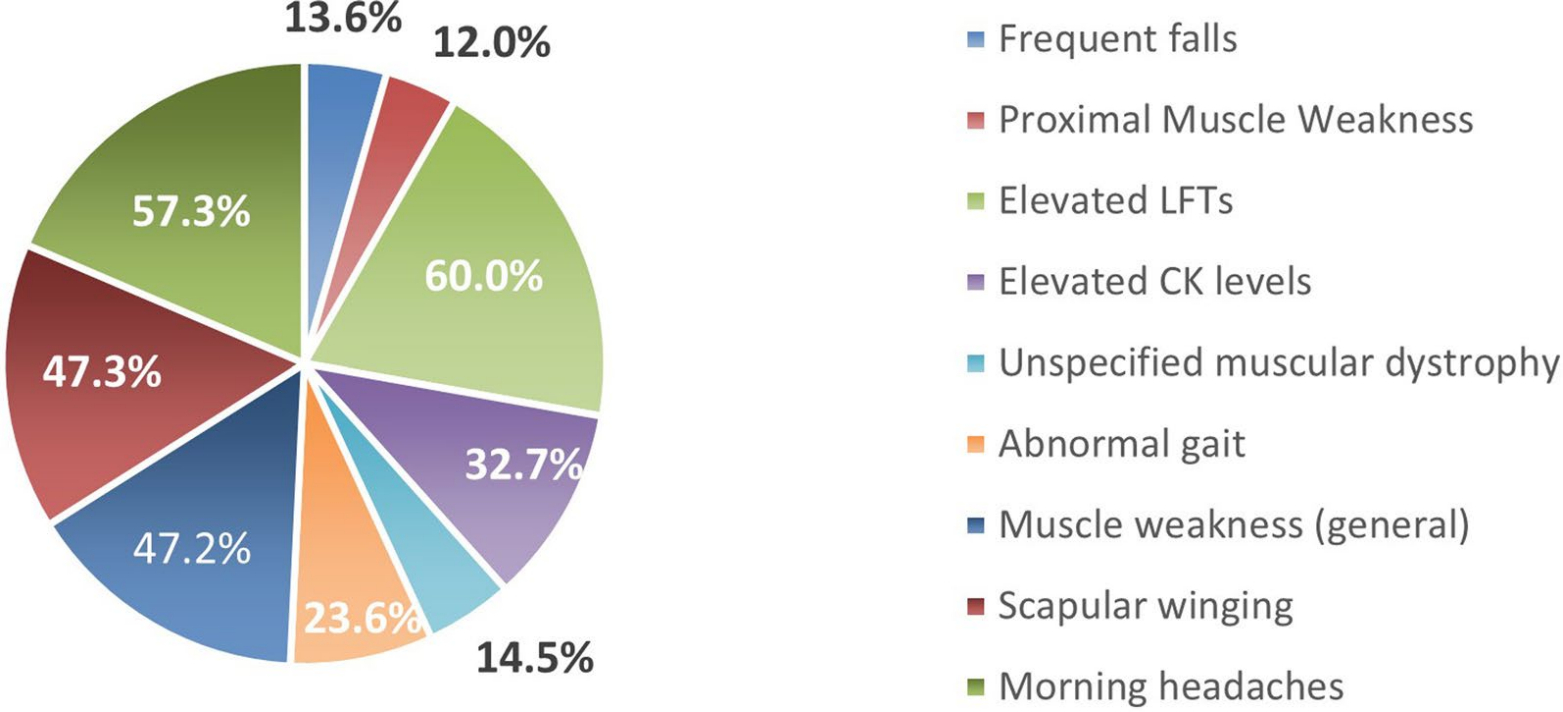
Symptoms seen most frequently in patients at risk for Late onset Pompe disease

The study team members then reviewed the EMR of 551 patients who had an ICD-10 code of interest and elevated ALT, AST, or CK values to look for any other explanations of the findings such as biliary atresia, non-alcoholic fatty liver disease, a stroke, or a cancer diagnosis. In the end, 110 “at-risk” patients scored > 100 points on the symptom-based LOPD scoring tool (Table 1, Fig. 1). The ruled out 441 patients did not have symptoms that scored high or supported LOPD as a probable diagnosis. In the 110 patients scoring more than 100 points,6 patients had a known diagnosis of LOPD (positive controls), 56 patients had a confirmed diagnosis of another genetic neuromuscular condition or phenocopy (see supplemental Table 1 for listing), 2 patients had already tested negative for LOPD, and 46 were considered to be at highest risk. The top symptoms seen in highest at-risk patients are delineated in Fig. 2.

These forty-six highest at-risk patients were contacted either directly or through their health care provider to participate based on presence of related symptoms. The specialties of healthcare providers contacted as part of the study included: neurology, cardiology, primary care, rheumatology, hematology, and orthopedics. All healthcare providers were given information on the scoring tool, the diagnosis, how to refer patients to genetics, and how to test for LOPD. Eight of the providers referred their patients to genetics for evaluation. Five patients chose to complete the consent process and being the genetic counseling and testing process.

Within the population of at-risk individuals, there were diverse responses to learning about a possible underlying genetic explanation for their symptoms. One patient reported that they had a muscle biopsy indicating glycogen storage disease. He believed he was also tested for LOPD and was affected by the condition, but did not wish further testing, follow-up, or treatment. A second patient declined testing and reported that his neurologist felt in his medical opinion it was not worth testing for LOPD. Three patients had pretest genetic counseling and were tested for decreased levels of acid-α-glucosidase as well as genetic testing for *GAA* pathogenic variants. Two of these individuals had both tested negative for PD on molecular testing with no variants in GAA found. One patient was identified with deficient acid-α-glucosidase and combined with symptoms suggested a diagnosis of LOPD. However, 2 pathogenic variants were not found on initial molecular testing and the patient was not interested in further evaluation. Due to COVID, chart review and scoring went quickly, but recruitment and patient willingness to complete testing was much slower than predicted (Fig. 1). The reasons for low acceptance rate of testing in the highest risk patients included: COVID exposure concerns, limited mobility or failing health, out of date contact information, and lack of interest.

From the population of healthcare providers given detailed information about LOPD and testing availability, one physician identified a new patient to his practice as ‘high-risk’ and tested them for LOPD. This 33-year-old patient was coded with ICD-10 G71.00, G71.09, G72.9, and R06.00. She also had an elevated CK. Her signs and symptoms included childhood onset generalized weakness that is worse in her lower33-year-olds, waddling gait, and asthma. She had been experiencing progressive weakness in lower extremities since 31 years of age. Based on her scoring at referral and her elevated CK level she would have been identified in our data search and scored > 100 points. The physician ordered a molecular neuromuscular panel and found 2 pathogenic variants in *GAA*. This patient was referred to Emory Genetics for monitoring and treatment.

## Discussion

This study illustrates that LOPD should be ruled out in patients with a diagnosis of proximal muscle weakness (G71.0), elevated liver functions tests, and/or elevated creatine kinase who do not already have a molecularly proven etiology. The use of a symptom-based scoring tool standardizes chart review and allows for faster identification of “at-risk” patients as well as pinpointing specific healthcare providers who would benefit from additional education on LOPD. The large set of patients with ICD-10 codes of interest and at least one lab value commonly abnormal in individuals with LOPD could be whittled down automatically in the future by adding negatively scoring ICD-10 coded diagnosis or symptoms related phenocopies to increase the specificity of the scoring tool.

Limitations of this study were that it was a small sample size, due in part to inability to contact many of eligible patients combined with many declining to participate. Reasons for declining varied: COVID concerns, limited mobility or failing health, and lack of interest. These limitations emphasize that a better method is needed to effectively convey the benefits and purpose of genetic testing for LOPD. Coordinating study participation with regular clinic visits and providers (such as in the López-Rodríguez study) and/or formal referral to genetics could increase participation in future studies [15]. Lastly, the retrospective nature of ICD-10 code search relies on correct input of appropriate codes. A potential patient could be missed if an ICD-10 code was not chosen or labs were collected during the time frame of the review.

Since the completion of this study, there have been significant advances in at-risk patient finding using big data sets, natural language processing, and artificial intelligence (AI) [16–18]. Several published tools have shown to be a more efficient way to use deidentified longitudinal health history data, positive/negative criteria, and predictive analytics to identify patients at greater risk for undiagnosed genetic conditions using medication history, prescription information, laboratory results, symptoms and signs, procedures, and diagnoses extracted from patient-level health care claims and electronic medical records [16–18]. These tools have had success identifying patients with genetic diseases and may contribute to increasing the diagnosis of LOPD; however, a keen clinician eye is still needed to validate the risk level of patients at the end of these processes.

Comparing published studies utilizing AI to identify patients at-risk for genetic conditions compared to the current study found several differences [16–18]. First, although the scoring tool algorithm used in this study is validated and optimized to identify patients living with LOPD, converting the scoring tool into an AI based tool may decrease the time required for review of patient datasets. Additionally, the number of patients reviewed could be broadened in numbers as an AI based tool can be run on a large medical records dataset as opposed to pulling data using one or two top symptoms or abnormal lab values. However, after increasing the efficiency and scoring using the AI based tool, a keen human clinical review is still needed to review all at-risk patients to rule out phenocopies and reduce false positives as seen in this study [19]. Next directions for identifying patients at-risk for LOPD should use a validated scoring tool or algorithm combined with big data sets of structured and unstructured data, natural language processing, artificial intelligence, and healthcare provider education to see if newer methods can efficiently and effectively decrease the time to diagnosis and treatment in patients with LOPD.

## Conclusions

LOPD should be considered when evaluating any patient with progressive muscular weakness and one lab value commonly abnormal in individuals with LOPD. This study confirms that a multipart symptom-specific scoring tool is a viable method of identifying patients at-risk for LOPD via retrospective EMR review. Identifying at-risk patients is the first step in diagnosing patients early in the disease presentation and allowing them the option of pursuing treatment that could help improve quality of life for this devastating disease.

## Supplementary Information

Additional file 1

## Data Availability

The authors declare that the key data supporting the findings of this study are available within the paper. The individual patient data cannot be shared openly to protect study participant privacy. Should any raw data files be needed in another format they are available from the corresponding author upon reasonable request.

## Abbreviations

AI: Artificial intelligence
CK: Creatine kinase
EHC: Emory Healthcare
EMR: Electronic medical record
ERT: Enzyme replacement therapy
FDA: Food and drug administration
ICD-9: International classification of diseases, ninth revision
ICD-10: International classification of diseases, tenth revision
GAA: Acid-α-glucosidase
LGH: Lafayette General Hospital System (Now Ochsner Lafayette General Medical Center)
LGMD: Limb girdle muscular dystrophy
LOPD: Late-onset Pompe disease
PD: Pompe disease
