# Supplementary material for: A study to identify individuals at risk to be affected by late-onset Pompe disease who had previously been given a non-specific or tentative diagnosis for their muscle weakness (Pompe PURSUE)
Source: Orphanet J Rare Dis. 2025 Jan 14;20:23. doi: 10.1186/s13023-024-03425-1 (PMC11731190; doi:10.1186/s13023-024-03425-1)
Supplement: Supplementary file 1 — Additional file 1 [file 13023_2024_3425_MOESM1_ESM.docx]

**Supplemental Data Table 2. Other Genetic Conditions Scoring 100 or above in Data Review**

| Becker muscular dystrophy  Bethlem myopathy  Charcot-Marie-Tooth disease  Duchenne muscular dystrophy  Emery Dreifuss muscular dystrophy  facioscapulohumeral muscular dystrophy  glycogen storage disease type 1A  limb-girdle muscular dystrophy types 1B, 1D, 2A, 2B, 2I, 2T  Miyoshi muscular dystrophy  myoadenylate deaminase deficiency  myotonic dystrophy  oculopharyngodistal myopathy  spinal muscular atrophy types II and III |
| --- |
|  |
